# The frequency of parent-reported motor coordination difficulties in children diagnosed with benign joint hypermobility syndrome

**DOI:** 10.1186/1546-0096-9-S1-O35

**Published:** 2011-09-14

**Authors:** C Bernie, SM Maillard

**Affiliations:** 1 Rheumatology Department, Great Ormond Street Hospital, London, UK

## Background

In recent studies, joint hypermobility in childhood has been associated with poorer activity participation and quality of life outcomes. In addition, evidence suggests that these children are more likely to present with motor co-ordination difficulties. Reduced motor competence in childhood has a range of reported long term adverse health outcomes including obesity, decreased self esteem, and secondary mental health issues such as anxiety and depression. This group therefore warrants further study, along with appropriate and timely interventions.

## Aims

To investigate the reported frequency and functional impact of motor-based difficulties in children presenting to a tertiary assessment clinic for biomechanical pain related to joint hypermobility.

## Methods

Data was collected from children and primary caregivers who attended a tertiary multidisciplinary assessment clinic between 2005 and 2009. Measures included recorded interview responses, range-of-motion testing, and functional assessment tools such as the Childhood Health Assessment Questionnaire (CHAQ). Data was collated and analyses completed using Welch’s T-tests for significance.

## Results

Out of 200 children assessed (40% male, age range 5-16 years), 172 children met criteria for Benign Joint Hypermobility Syndrome (BJHS). Of these, clumsiness was reported in 50.6%, 46.5% reported coordination difficulties, and 34.9% identified poor ball skills. In addition, 22.7% reported all three issues, indicating that almost a quarter of this group present with widespread motor-based deficits. This is considerably higher than recent published estimates for the United Kingdom, reporting that 4% of children have moderate coordination difficulties. Children with BJHS and reported coordination difficulties also presented with higher CHAQ scores (p < .001) than other study participants.

## Conclusions

Children presenting with joint hypermobility frequently report motor coordination dysfunction. Within the BJHS group, children with reported coordination issues present with greater impairment in activities of daily living. Services should support such children to improve participation, independence, and quality of life, whilst aiming to prevent the onset of secondary health problems.

